# Proposed equation between flexor carpi radialis H-reflex latency and upper limb length

**Published:** 2015-01-05

**Authors:** Saeid Khosrawi, Parisa Taheri, Seyed Hasan Hashemi

**Affiliations:** Department of Physical Medicine and Rehabilitation, Faculty of Medicine, Isfahan University of Medical Sciences, Isfahan, Iran

**Keywords:** H-reflex, Normal Volunteers, Arm

## Abstract

**Background:** H-reflex is a valuable electrophysiological technique for assessing nerve conduction through entire length of afferent and efferent pathways, especially nerve roots and proximal segments of peripheral nerves. The aim of this study was to investigate the relation between normal values of flexor carpi radialis (FCR) H-reflex latency, upper limb length and age in normal subjects, and to determine whether there is any regression equation between them.

**Methods: **By considering the criteria of inclusion and exclusion, 120 upper limbs of 69 normal volunteers (68 hands of 39 men and 52 hands of 30 women) with the mean age of 39.8 ± 11.2 years participated in this study. FCR H-reflex was obtained by standard electrodiagnostic techniques, and its onset latency was recorded. Upper limb length and arm length were measured in defined position. The degree of association between these variables was determined with Pearson correlation and linear regression was used for obtaining the proposed relations.

**Results: **Mean FCR H-reflex latency was found to be 15.88 ± 1.27 ms. There was a direct linear correlation between FCR H-reflex latency and upper limb length (r = 0.647) and also arm length (r = 0.574), but there was no significant correlation between age and FCR H-reflex latency (P = 0.260). Finally, based on our findings, we tried to formulate these relations by statistical methods.

**Conclusion:** We found that upper limb length and arm length are good predictive values for estimation of normal FCR H-reflex latency but age, in the range of 20-60 years old, has no correlation with its latency. This estimation could have practical indications in pathologic conditions.

## Introduction

One type of electrophysiological late responses was described by Hoffmann, hence it is named as H-reflex.^1^ This reflex is an electrical analog of the monosynaptic stretch reflex provoked by bypassing the muscle spindle and is obtained in only a few muscles in normal adults and can be elicited by submaximal stimulation of the nerves.^2^^,^^3^ The stimulus travels along the Ia fibers and through the dorsal root ganglion, then it is spread to the anterior horn cell, which fires it down along the alpha motor axon to the muscle. It can be simply attained in soleus muscle (with stimulation of posterior tibial nerve at popliteal fossa), flexor carpi radialis (FCR) muscle (with stimulation of median nerve at elbow), and quadriceps muscle (with femoral nerve stimulation).^4^

H-reflex is a valuable electrodiagnostic technique for assessing nerve conduction through entire length of afferent and efferent pathways, especially at proximal segment of peripheral nerve, and also for evaluating neurophysiological changes in compromised nerve roots and efficacy of some of nonsurgical managements on patients with radiculopathy.^5^^-^^7^ While studying H-reflex in clinical situations such as evaluation of patients suspected to radiculopathies, electromyographers usually use the parameter of latency for their interpretations and its amplitude less likely get attention because of wide variability. H-reflex is usually present in normal subjects, but this is not permanently correct. Symmetrically absent H-reflexes are not essentially abnormal and percentage of absent responses increases inelderly.^8^ The most common parameter of H-reflex used, is prolonged onset latency and/or absence of H-reflex on affected side.^9^^-^^15^

Many studies have found that H-reflex of lower limbs is strongly correlated with both age and leg length. Ghavanini et al. studied the role of various constitutional factors influencing H-reflex latency and among them limb length was the only variable strongly correlated with H-reflex latency.^16^ A nomogram and regression equation for obtaining individual optimal soleus H-reflex latencies have been represented by Braddom and Johnson.^17^^,^^18^

Regarding H-reflex in upper limbs there are very limited studies published in literature.^5^^,^^12^^-^^14^ To the best of our knowledge, there are only two published articles by Schimsheimer et al.^12^ and Schimsheimer et al.^13^, which have introduced equations for estimation of optimal FCR H-reflex latency but these equations have not get popularity due to their complexity.

According to importance of FCR H-reflex latency in diagnosis of C6-C7 radiculopathies and limited studies in this field, we decided to perform a study in order to investigate relation between FCR H-reflex latency, upper limb length, arm length-a parameter of proximal upper limb length that has not been considered in previous studies-and age in normal Iranian population. In addition, we tried to find a practical formula for calculating and estimating optimal FCR H-reflex latency based on these parameters.

## Materials and Methods

This cross-sectional study was carried out from October 2013 to April 2014 among 120 upper limbs of healthy volunteers with an age range of 20-60 years. Samples were selected with a conventional method from volunteers and patients referred to academic electrodiagnostic centers, after explaining the procedure and taking written consent.

All of the participants were persons who had neither signs nor symptoms of neurologic abnormalities of upper extremities in their history and physical examination. The subjects who had any history of hereditary polyneuropathies (e.g., Charcot-Marie-Tooth), acquired polyneuropathies (e.g., diabetic polyneuropathy), any scar formation or history of fracture in their upper limbs, including the sites of stimulation or recording were excluded from our research.

**Figure 1:**
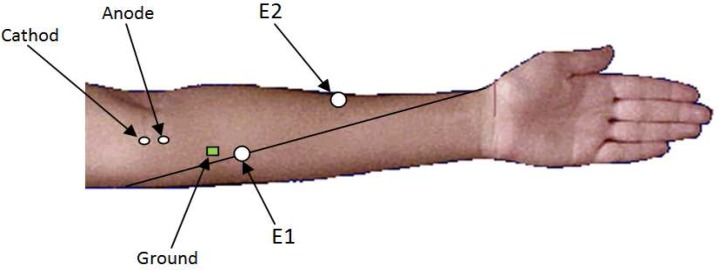
The placement of electrodes in recording of flexor carpi radialis (FCR) H-reflex

The procedure was done in a setting with mean room temperature of 25 °C and skin temperature of 32-34 °C while the subject lying supine.^19^ All electrodiagnostic tests were performed with Cadwell Sierra Wave electromyography machine. Surface stimulating bar electrode with 0.5 cm in diameter and cathode-anode distance of 2 cm was applied longitudinally on median nerve in antecubital fossa with cathode proximal to anode. Surface E-1 recording electrode was positioned over belly of FCR muscle located one third of distance between medial epicondyle and radial styloid and an E-2 recording electrode was placed over brachioradialis muscle. As a ground, a metal electrode was applied on skin of forearm just proximal to E-1 electrode (Figure 1).

The electrodes were not removed until the whole experiment was completed to ensure exact placement and consistent results.^20^ A pulse width of 0.5-1 ms was delivered at a frequency of one pulse per 2-3 s to median nerve. Sweep speed of instrument was 5 ms/div with sensitivity between 0.5 and 1 mV/div. Onset latency of H-reflex was measured from stimulus artifact to the first deflection from baseline. Stimulating electrode placement was considered acceptable when maximum H-reflex could be elicited with minimal or no M response and presence of H-reflex verified by increasing the stimulus intensity and observing a disappearance of the response and replacement by an F-wave. At least five H-responses were studied for analysis to ensure its reproducibility.

Upper limb length was measured in centimeters from C6 spinous process to tip of the third digit while arm was in 90°abduction, elbow in full extension, forearm in pronation and digits in the extension. Arm length was also measured in the same position from C6 spinous process to tip of olecranon.

Data analysis was performed by SPSS for Windows (version 16, SPSS Inc., Chicago, IL, USA). P < 0.050 accepted as statistically significant. Results are presented as means ± standard deviation. Independent T-test was used for analyzing sex-related differences and degree of association between variables was determined with Pearson correlation. Linear regression was used for obtaining proposed relations.

## Results

Based on inclusion and exclusion criteria, 120 upper limbs of 69 healthy subjects (68 hands of 39 men and 52 hands of 30 women) participated in this study. The study was performed on both hands of 51 subjects but it was carried out in one hand of each of other 18 participants because of different problems (10 had symptoms of paresthesia, 5 had positive Tinel or Compression signs and 3 had scar formation on their other hand).

The subjects’ mean upper limb length was 88.5 ± 3.6 cm with a range of 81.3-95.7 cm and mean arm length were 45.8 ± 2.3 cm with a range of 41.3-50.3 cm. Mean FCR H-reflex latency was 15.88 ± 1.27 ms with a range of 13.34-18.42 ms.

Independent T-test analysis revealed statistically significant differences between men and women in FCR H-reflex latency, upper limb length and arm length which are shown in table 1. 

**Figure 2:**
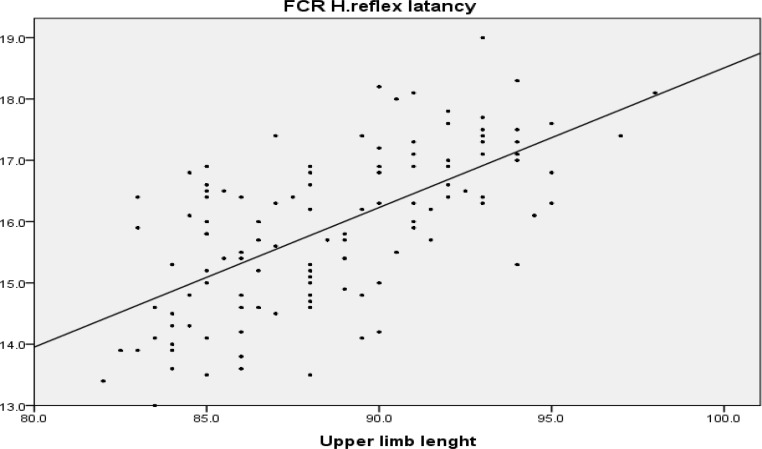
Regression line between flexor carpi radialis H-reflex and upper limb length

**Figure 3:**
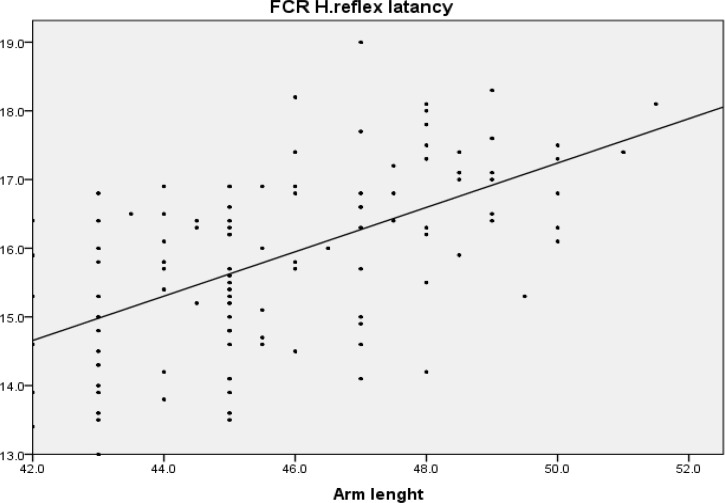
Regression line between flexor carpi radialis H-reflex and arm length

Pearson correlation shows that there is a direct linear correlation between upper limb length and FCR H-reflex latency(r = 0.647, P < 0.001) and also between arm length and FCR H-reflex latency (r = 0.574, P < 0.001), but there was no significant correlation between age and FCR H-reflex latency (P = 0.260). All correlation coefficients are shown in table 2. The regression XY chart depicted in figures 2 and 3.

Based on linear regression analysis the following formulae for the prediction of optimal FCR H-reflex latency were obtained:

FCR H-reflex latency (ms) = 0.23 × upper limb length (cm) –4.3

FCR H-reflex latency (ms) =0.32 × arm length (cm) + 1.1.

**Table 1:** Independent T-test analysis for difference of variable means among men and women

**Variable**	**Mean ± SD**		**P**
	**Men**	**Women**	
FCR H-reflex latency (ms)	16.3 ± 1.2	15.4 ± 1.2	< 0.001
Upper limb length (cm)	90.4 ± 3.1	86.0 ± 2.6	< 0.001
Arm length (cm)	47.0 ± 2.0	44.3 ± 1.6	< 0.001

FCR: Flexor carpi radialis; SD: Standard deviation

**Table 2:** Pearson correlation coefficients between different variables

**Variable**	**Upper limb length**	**Arm length**	**Age**	**FCR H-reflex latency**
Upper limb length Pearson correlation	1.000	0.913**	-0.248**	0.647**
Arm length Pearson correlation	0.913**	1.000	-0.213*	0.574**
Age Pearson correlation	-0.248**	-0.213*	1.000	0.103*
FCR H-reflex latency Pearson correlation	0.647**	0.574**	0.103*	1.000

FCR: Flexor carpi radialis; That direct linear correlation between upper limb length and FCR H-reflex latency is stronger than correlation between arm length and FCR H-reflex latency; also it shows there is no significant correlation between age and FCR H-reflex latency

* Correlation is significant at the 0.05 level;

** Correlation is significant at the 0.01 level;

FCR: Flexor carpi radialis

FCR: Flexor carpi radialis

## Discussion

Several studies have determined that FCR H-reflex is a valuable supplement to conventional conduction studies for diagnosis of upper limb pathologies.^12^^-^^15^ In the present study, we investigated the correlation between different variables and FCR H-reflex latency and also defined two formulae to predict optimal FCR H-reflex latency based on upper limb length and arm length.

Consistent with the results of Ongerboer et al. we didn’t find any significant relationship between FCR H-reflex latency and age.^21^ However, Schimsheimer et al. found that it may be used to predict FCR H-reflex latency and inter-latencytime.^12^ Based on pathophysiologic descriptions H-reflex latency would be expected to proportionally prolong in elderly because of its pathway seems to be affected by several age-related changes, involving both interneurons and the afferent and efferent tracks.^3^^,^^9^^,^^16^^,^^22^ However maybe the effect of age is much less apparent on these parameters in subjects under 60 years-old as our subjects were in the range of 20-60 years old and the age-related changes are more important in older subjects.

H-reflexes have a long pathway that may be influenced by multiple factors and length of the limb may have a significant influence on its latency.^4^ Based on anatomical pathway of H-reflex loop, this correlation between either thigh length and soleus H-reflex latency or upper limb length and FCR H-reflex latency are expectable. Several studies have investigated the relation between leg length, height and soleus H-reflex latency and found significant correlation.^17^^,^^18^^,^^23^^,^^24^ Although a recent study did not find any relation between individual thigh length and soleus H-reflex latency.^25^ Another found that in normal subjects regression equations demonstrate latencies of FCR and soleus H-reflexes can be predicted from limb length but equally accurately from body height.^14^ Limited studies have shown the effect of upper limb length on FCR H-reflex latency,^12^^,^^13^ but arm length has not been investigated before. In this study, we found a significant correlation between FCR H-reflex latency and both upper limb length and arm length; however this correlation was stronger between FCR H-reflex latency and upper limb length. This could be attributed to the fact that upper limb length include forearm region too, therefore FCR muscle inherent motor parameters can influence more apparently on H-reflex latency. However, more studies are needed to examine and compare the effects of these parameters on the FCR H-reflex latency.

As our results show, there are significant differences between FCR H-reflex latency as well as upper limb length and arm length among men and women. Thus besides intrinsic differences between characteristics of nerve fibers and muscles of males and females, it may conclude that upper limb and arm lengths have had significant effects on FCR H-reflex latency. Of course, other parameters such as arm/forearm diameter may have important influences that could be evaluated in future researches.

Two studies performed in 1985 and 1987 investigated clinical application of FCR H-reflex latency, and they found multiple formulae to estimate it based on different variables such as age, upper limb length and body height.^12^^,^^13^ For instance one of these formulae is:

FCR H-reflex latency=1.29 + 0.01630 × limb length + 0.0270 × age ± 0.83

In comparison with our formula, it is obvious that for clinical application, they are not as much practical as ours. Additionally, they involved age and body height in their estimation but we did not. However the clinical usage and interpretation of the found formulae in this paper, should be explored by more researches.

While many segmental and supra-segmental parameters related to both physical and mental state of the individual can influence different parameters of H-Reflex and hence it seems that additional aspects can also be critical on prediction of FCR H-reflex latency.^8^^,^^26^^,^^27^ These parameters should be considered and reviewed for better prediction which may include complete muscle relaxation and free of anxiety, effects of facilitatory maneuvers and antagonist muscle stimulation.

The findings of this study were interpreted in the light of previous studies, and one can say that this study will help to further determine various neurological diseases with different limb lengths. This research will also help in making a normative data for diagnosing various neurological diseases.

## Conclusion

The purpose of this study was to assess the relation between FCR H-reflex latency, upper limb length, arm length and age in healthy subjects. We found that although age has no correlation with FCR H-reflex latency but upper limb length and arm length are predictive values for estimating its latency.

## Notes

**How to cite this article: **Khosrawi S, Taheri P, Hashemi SH. Proposed equation between flexor carpi radialis H-reflex latency and upper limb length. Iran J Neurol 2015; 14(1): 41-6.
